# Metabolomic Analysis Reveals the Therapeutic Effects of MBT1805, a Novel Pan-Peroxisome Proliferator-Activated Receptor Agonist, on α-Naphthylisothiocyanate-Induced Cholestasis in Mice

**DOI:** 10.3389/fphar.2021.732478

**Published:** 2021-10-29

**Authors:** Chang Wang, Fei Peng, Bohua Zhong, Ying Shi, Xiaomei Wang, Xueyuan Jin, Junqi Niu

**Affiliations:** ^1^ Department of Hepatology, The First Hospital of Jilin University, Changchun, Jilin, China; ^2^ Key Laboratory of Zoonosis Research, Ministry Education, Changchun, Jilin, China; ^3^ Beijing JK HuaYuan Med Tech Company LTD, Beijing, China; ^4^ International Center for Liver Disease Treatment, Fifth Medical Center of China PLA General Hospital, Beijing, China

**Keywords:** cholestasis, α-naphthylisothiocyanate, MBT1805, peroxisome proliferator-activated receptor, agonist

## Abstract

**Background and Aims:** Therapeutic drugs that are used to treat cholestatic liver disease are limited; however, the results of clinical trials on primary biliary cholangitis treatment targeting peroxisome proliferator-activated receptors (PPARs) are encouraging. In this study, we aimed to identify the effects of MBT1805, a novel balanced PPARα/γ/δ agonist, on cholestasis induced by α-naphthylisothiocyanate (ANIT) and elucidate the underlying mechanisms through untargeted and bile acid-targeted metabolomic analysis.

**Methods:** Levels of serum biochemical indicators (transaminase, aspartate transaminase, alkaline phosphatase, and total bilirubin) and liver histopathology were analyzed to evaluate the therapeutic effects of MBT1805 on ANIT-induced cholestasis in C57BL/6 mice. Untargeted and bile acid-targeted metabolomic analysis of liver tissues was performed using ultrahigh-performance liquid chromatography-triple quadrupole mass spectrometry (UPLC-MC/MC). qRT-PCR and Western blot analysis were carried out to measure the expression of key enzymes and transporters regulating bile acid synthesis, biotransformation, and transport.

**Results:** MBT1805 significantly improved abnormal levels of liver biochemical indicators and gallbladder enlargement induced by ANIT. Histopathological analysis showed that MBT1805 effectively relieved ANIT-induced necrosis, vacuolation, and inflammatory infiltration. Untargeted metabolomic analysis identified 27 metabolites that were involved in the primary biliary acid biosynthesis pathway. In addition, bile acid-targeted metabolomics showed that MBT1805 could alleviate the abnormal bile acid content and composition induced by ANIT. Furthermore, qRT-PCR and Western blot results confirmed that MBT1805 could effectively regulate bile acid synthesis, biotransformation, and transport which helps relieve cholestasis.

**Conclusions:** MBT1805 is a potential candidate drug for cholestasis, with a balanced PPARα/γ/δ activation effect.

## Introduction

Impaired bile flow and accumulation of toxic bile acids are the main causes of cholestasis injury, which can lead to liver tissue destruction, inflammation infiltration, and subsequent fibrosis and cirrhosis (Arrese and Trauner, 2003). Patients who are not properly treated may develop end-stage liver disease. Once the end-stage liver disease develops, liver transplantation is the only treatment option since effective drugs are still lacking.

Primary biliary cholangitis (PBC) and primary sclerosing cholangitis (PSC) are the main chronic cholestasis disease. Ursodeoxycholic acid (UDCA) was the only treatment for PBC until the approval of obeticholic acid (OCA), which is approved as a monotherapy or combined with UDCA (Trivedi et al., 2016). Approximately 40% of patients with PBC have an inadequate response to UDCA treatment (Corpechot et al., 2011), whereas OCA has some adverse effects such as pruritus, fatigue, and discomfort (Kowdley et al., 2018). Although dose-dependent reductions in alkaline phosphatase (ALP) levels were observed in trials of PSC treatment with UDCA, long-term effects, such as cirrhosis manifestation, acute decompensation of cirrhosis, liver transplantation, and death events, need further assessment (Olsson et al., 2005).

Nuclear receptors (NRs) effectively regulate gene expression related to the elimination of toxic biliary constituents accumulating in cholestasis and are potential targets of PBC treatment (Halilbasic et al., 2013). Peroxisome proliferator-activated receptors (PPARs) belong to the NR family and comprise three isoforms: PPARα, PPARβ/δ, and PPARγ. PPARα agonists can treat hyperlipidemia, whereas PPARγ agonists effectively manage type 2 diabetes (Hong et al., 2018). However, some adverse effects have restricted the use of PPAR agonists, such as weight gain and fluid retention. Dual or pan-PPAR agonists are expected to reduce the side effects of single receptor activation. For instance, elafibranor, a dual PPARα/δ agonist, has completed phase-2 clinical trials and has reached the primary and secondary endpoints. Fibrates, such as fenofibrate and bezafibrate, have also been used to treat PBC with encouraging outcomes (Honda et al., 2013; Corpechot et al., 2018; Ghonem et al., 2020).

MBT1805 is a novel drug candidate built on resveratrol scaffolds and it is the most balanced PPAR-α/γ/δ agonist with moderate activity (EC50: 8.46, 11.94, and 11.5 μM, α/γ/δ, respectively). The synthesis of MBT1805 has been elaborately described in an early study (Li et al., 2010). Previous studies showed that activation of PPARα alleviates cholestasis injury caused by α-naphthylisothiocyanate (ANIT) (Fang et al., 2017; Dai et al., 2017; Zhao et al., 2017; Dai et al., 2018). The animal model of cholestasis induced by ANIT used in this research was widely used in previous studies, especially in the exploration of novel treatment drugs for cholestasis (Pollheimer and Fickert, 2015; Fang et al., 2017; Wang H. et al., 2017; Wu et al., 2017).

Metabolomics focuses on the study of endogenous small molecules with relative molecular weights of <1,000 Da (Nicholson et al., 1999), mainly using nuclear magnetic resonance (NMR) and mass spectrometry. Untargeted metabolomics intends to comprehensively analyze all measurable metabolites that are widely used in mechanism analysis and biomarker discovery, whereas targeted metabolomics focuses on the analysis of specific metabolic components that are suitable for pharmacokinetic studies (Roberts et al., 2012; Gertsman and Barshop, 2018).

In this study, we aimed to evaluate the role of MBT1805 for treating ANIT-induced cholestatic liver injury and revealed the possible therapeutic mechanisms through untargeted and bile acid-targeted metabolomics.

## Materials and Methods

### Animals and Treatments

Six-week-old male C57BL/6 mice were purchased from Charles River Laboratories (Beijing, China). Mice were housed in a breeding room at the Experimental Animal Center of the Translational Medical Research Institute, First Hospital of Jilin University, Changchun, China. They were maintained at 25°C ± 1 C, 60%–70% humidity, and a light/dark cycle of 12 h with libitum access to feed and water. After adaptive feeding of normal growth chow from Xietong (Jiangsu, China) for 1 wk, mice were divided into groups: control, no treatment (*n* = 10); model, treatment only with ANIT (*n* = 10); UDCA-60, treatment with 60 mg kg^−1^ UDCA and ANIT (*n* = 10). The dose of UDCA was referred to the research of Wang et al. (Wang L. et al., 2017): MBT1805-10, treatment with 10 mg kg^−1^ MBT1805 and ANIT (*n* = 10); MBT1805-20, treatment with 20 mg kg^−1^ and ANIT (*n* = 10); and MBT1805-30, treatment with 30 mg kg^−1^ MBT1805 and ANIT (*n* = 10). UDCA and MBT1805 were administrated by gavage for 7 days. All mice were euthanized 24 h after the administration of 75 mg kg^−1^ ANIT. ANIT and MBT1805 were supplied by Beijing, JK HuaYuan Med Tech Company LTD (Beijing, China).

### Biochemical Analysis

Blood samples were collected from the heart, placed into 4 ml centrifuge tubes, and left at 25°C for 30 min. Serum samples were centrifuged at 4,000 rpm for 10 min at 4°C. Serum alanine aminotransferase (ALT), aspartate aminotransferase (AST), ALP, and total bilirubin (Tbil) levels were measured using assay kits (Nanjing Jiancheng Bioengineering Institute, Nanjing, China), according to the manufacturer’s instructions. Triglyceride and total protein were detected using assay kits (Nanjing Jiancheng Bioengineering Institute, Nanjing, China).

### Histopathological Evaluation

The liver tissue was cut into several parts, one of which was fixed with 4% paraformaldehyde solution. Fixed liver tissues of the above experiments were dehydrated in a serial concentration of alcohol and xylene followed by paraffin embedding. 4 μm of each sample was cut and stained with hematoxylin and eosin. Other liver tissues were stored in liquid nitrogen for subsequent RNA, protein extraction, and metabolomic analysis.

### Metabolomics Study

#### Sample Preparation and Operation Conditions

Liver tissue samples stored in liquid nitrogen were thawed on ice. For untargeted metabolomics, 50 ± 2 mg of tissue was mixed with cold steel balls and homogenized at 30 Hz for 3 min. Therefore, 1 ml of 70% methanol with standard internal extract (Metware Biotechnology, Wuhan, China) was added to the homogenized tissue and mixed by stirring for 5 min. The supernatant was collected after centrifugation at 12,000 rpm for 10 min at 4°C and stored at −20°C overnight. After centrifugation at 12,000 rpm for 3 min at 4°C, 200 μl of the supernatant was collected for ultrahigh performance liquid chromatography-triple quadrupole mass spectrometry (UPLC-MC/MC).

For bile acid-targeted metabolomics, 20 mg of tissue was ground with a ball mill, mixed with 200 μl methanol, and then stored at −20 C to precipitate protein. Extracts were dried by evaporation and reconstituted in 100 μl of 50% methanol (v/v) for UPLC-MC/MC. The instrumentation and operation conditions are described in the appendix, section1 (Supplementary Appendix S1). To identify metabolites, we used the self-built widely targeted metabolome database MWDB (Metware Biotechnology, Wuhan, China).

#### Metabolomic Data Analysis

Principal component analysis (PCA) and partial least squares discrimination analysis (PLS-DA) of metabolomic data were performed using SIMCA-P 14.1 (Umetrics, Umea, Sweden). All variables were mean-centered and scaled to a Pareto variance before PCA. Variable influence on the projection (VIP) was used to identify variables responsible for group separation, and only those with VIP ≥2.0 were selected for further analysis. Compounds with significant changes among groups (*p* < 0.05) were considered biomarkers and used for enrichment and pathway analysis with MetaboAnalyst 5.0.

### Quantitative Analysis of Hepatic Bile Acids

UPLC-MS/MS was used to detect the levels of various bile acids in mouse livers. The instrumentation and operation conditions are described in the appendix, section 2 (Supplementary Appendix S2).

### qRT-PCR

All RNA from liver tissues was isolated using the Promega SV Total Isolation System (Promega, Madison, WI, United States) and quantified using a microplate reader. cDNA was synthesized using the PrimerScript first strand cDNA Synthesis Kit (Takara, Shiga, Japan). The mRNA expressions of key enzymes (CYP27A1, CYP7A1, and CYP8b1), transport (NTCP, bile salt excretion pump (BSEP), MRP2, MRP3, and MRP4), and metabolic enzymes (CYP) were detected using the Agilent Mx3005 P Real-Time PCR System (Agilent, Santa Clara, CA, United States) with the SYBR Green PCR kit. The thermal conditions were as follows: 95°C for 60 s, followed by 45 cycles at 95°C for 15 s and 60°C for 60 s. β-Actin was used as an internal control. All primers used for qRT-PCR were synthesized by Sangon Biotech (Shanghai, China) and are presented in appendix, section 3 (Supplementary Appendix S3).

### Western Blot Analysis

Protein extracted from rat liver tissue was prepared using radio-immunoprecipitation assay lysis buffer (Beyotime, Shanghai, China). Protein concentrations were detected using a bicinchoninic acid protein assay (BCA) kit. Protein extract was loaded and electrophoresed through sodium dodecyl sulfate-polyacrylamide gel electrophoresis (SDS-PAGE) and transferred onto a polyacrylamide difluoride membrane (Immobilon®-P^SQ^). After blocking with 5% skim milk in Tris-buffered saline containing 0.1% Tween-20 (TBST) for 1 h at room temperature, membranes were incubated overnight at 4 C with primary antibodies and washed with 0.1% TBST Tris-buffered saline. The membranes were subsequently incubated with HRP-conjugated secondary antibodies for 30 min and then washed with 0.1% TBST Tris-buffered saline. Then, enhanced chemiluminescent kit (ECL) (New Cell & Molecular Biotech Co., Ltd) was used to detect the protein-antibody complexes using ChemiDoc XRS+ system (Bio-Rad, United States). All primary and secondary antibodies used in this experiment were supplied in appendix, section 4 (Supplementary Appendix S4).

### Statistical Analysis

Data were expressed as means ± standard error. Statistical differences among groups were determined by Welch and Brown-Forsythe one-way analysis of variance and the post hoc test of Dunnett T3. Statistical analysis was performed by SPSS 23.0. The statistical significance was set at *p* < 0.05.

## Results

### Biochemical Analysis and Pathological Assessment

**FIGURE 1:**
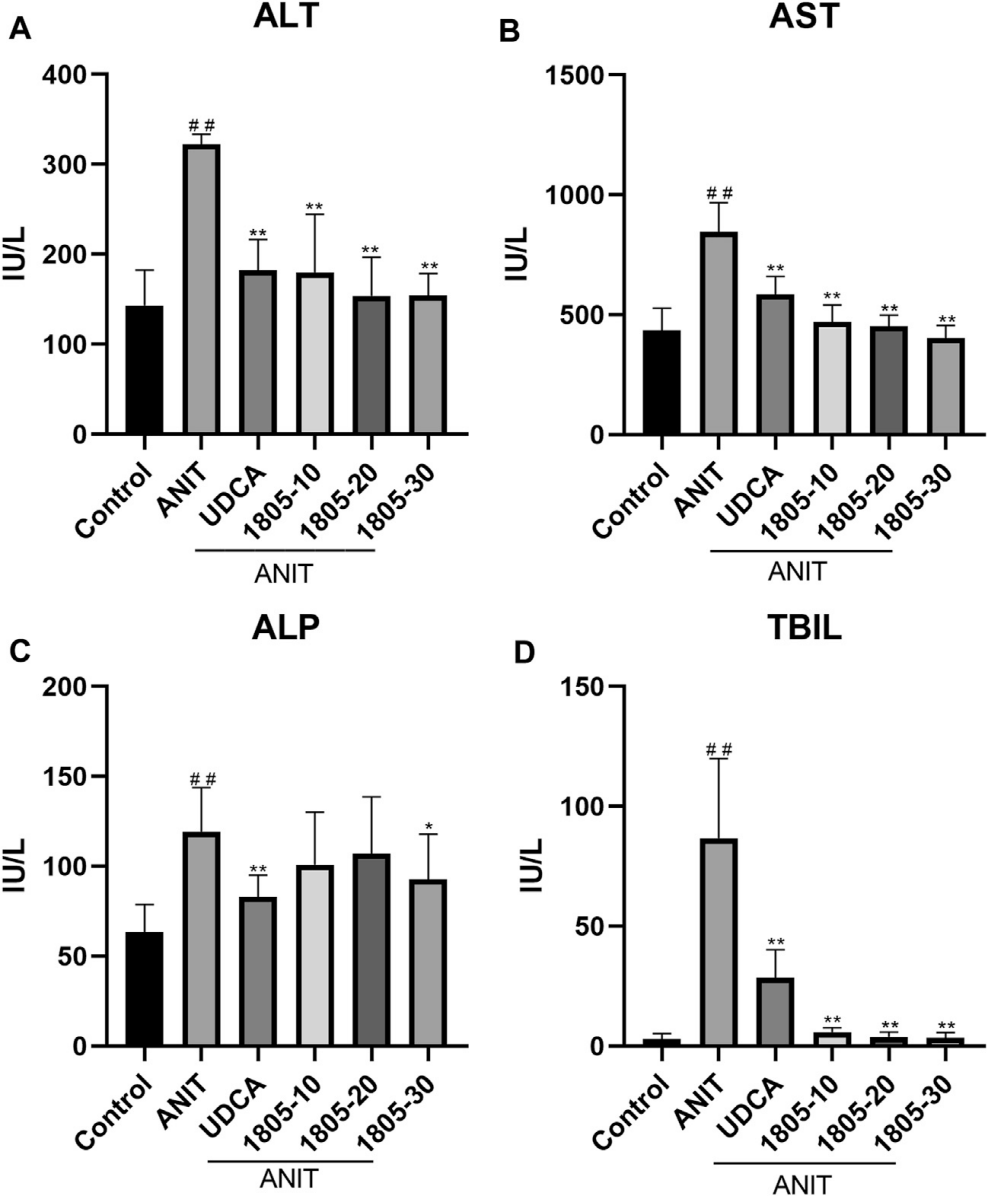
Serum parameters. **(A)** Alanine transaminase (ALT); **(B)** Aspartate aminotransferase (AST); **(C)** Alkaline phosphatase (ALP); **(D)** Total bilirubin (Tbil). #*p* < 0.05 and ##*p* < 0.01 compared with the control group; *p < 0.05 and **p < 0.01 compared with the ANIT group.

**FIGURE 2:**
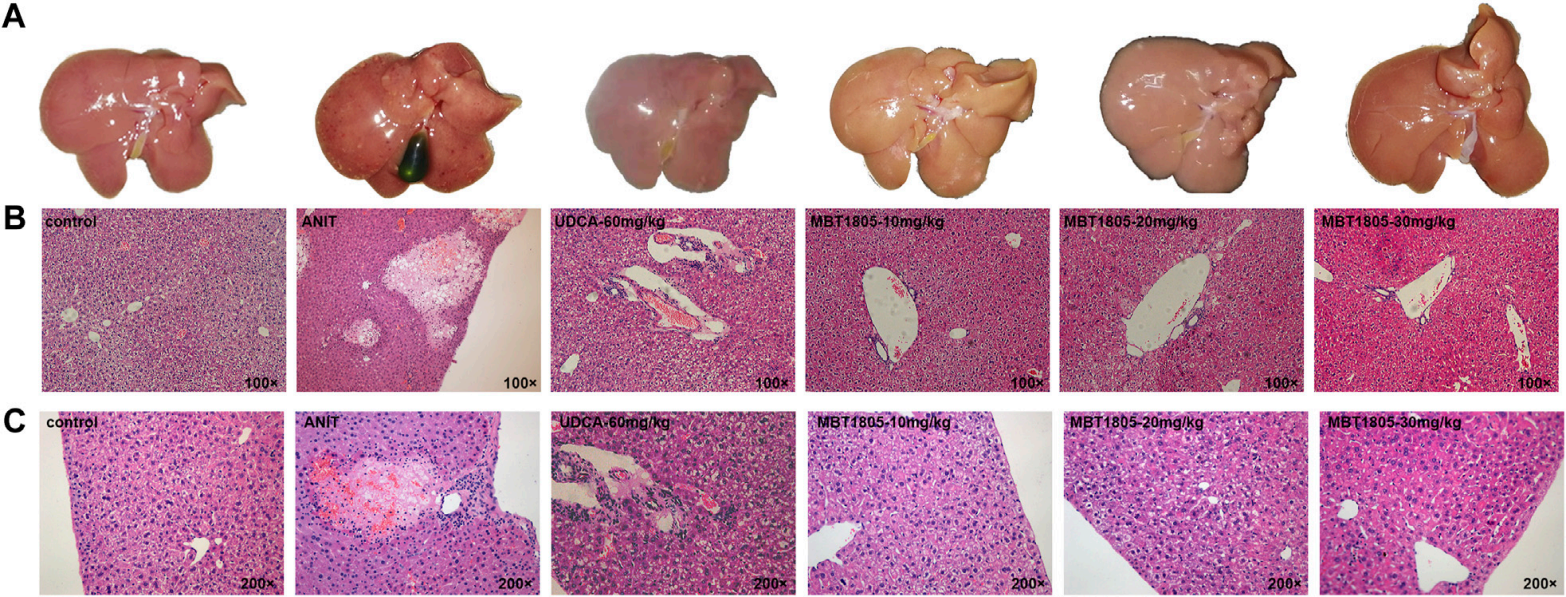
Appearance of gallbladders and pathology of liver tissues. **(A)** Appearance of gallbladders in different groups. **(B)** Pathology of liver tissues with magnification of 100×. **(C)** Pathology of liver tissues with magnification of 200×.

Compared with the control, all serum indicators were markedly increased in the model group (Figures 1A–D). UDCA and MBT1805 were effective in reducing ALT, AST, and Tbil, and MBT1805 was better. In addition, ALT and AST levels significantly reduced in the MBT1805-treated group in a dose-dependent manner (Figures 1A,B). Reduction of ALP was only observed in high dose of MBT1805 compared with the model group. ALP level was lower in the UDCA-treated group than that of the MBT1805-treated group (Figure 1C). Compared with the control, the gallbladder of the model was abnormally enlarged and filled with bile acids, whereas that of the MBT1805-treated groups appeared normal without any liver tissue congestion (Figure 2A).

Histopathological evaluation revealed significant hepatic tissue changes, such as necrosis, inflammatory infiltration, and vacuolization, in the model. In contrast, the UDCA- and MBT1805-treated groups exhibited less inflammatory infiltration and no necrotic tissues (Figures 2B,C).

### Multivariate Statistical Analysis

**FIGURE 3:**
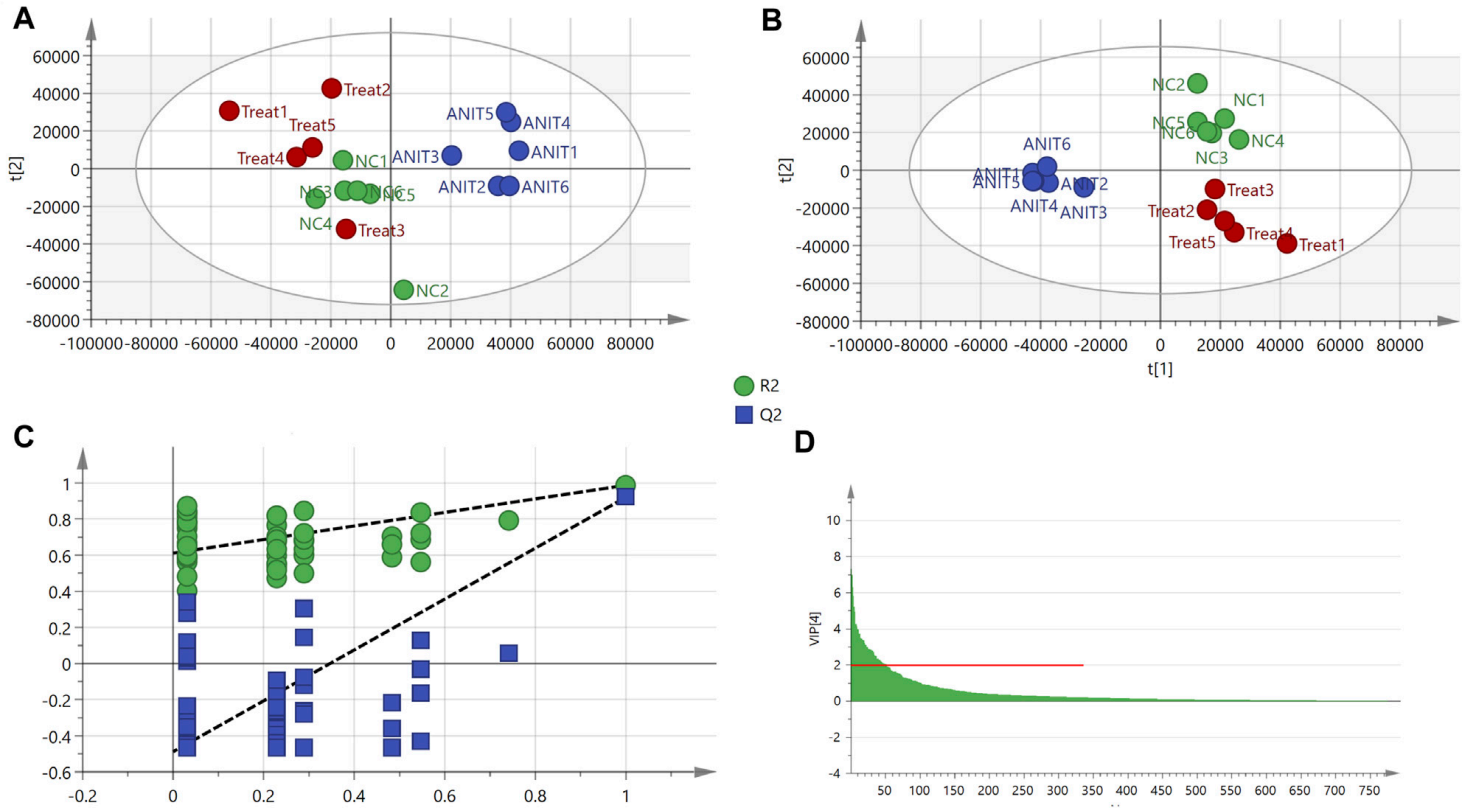
PCA and PLS-DA. **(A)** PCA score of control (*n* = 6), ANIT (*n* = 6), and MBT1805 30 mg/kg treatment (treat, *n* = 5). **(B)** Score plots of PLS-DA. **(C)** PLS-DA model maturation test with 100 (Q2 intercept =0.048). **(D)** VIP values with threshold, VIP ≥ 2.

In total, 793 metabolites were recognized after the combination of ESI^+^/ESI^−^ detection signals. PCA identified differences among the control, model, and MBT1805-30 groups (Supplementary Appendix S5). We found that the sample named treat6 in the MBT1805-treated group was an outlier (confidence interval, >95%) in PCA and was, thus, removed from the following analysis. The clustering of the model was significantly different from that of the control or MBT1805-30; however, no differences were identified between the latter two groups (Figure 3A). Therefore, further analysis is needed. PLS-DA separated the control, model, and MBT1805-treated groups. R^2^Y and Q^2^ were 0.906 and 0.832, respectively (Figure 3B), indicating the high accuracy of predictions. A permutation test was performed with 100 iterations. The validation plots had an intercept of Q^2^ < 0.5 (Figure 3C), and VIP ≥2.0 was used as a threshold to identify potential metabolites (Figure 3D).

### Identification of Potential Metabolites

Among the 793 detected compounds, 52 were selected from the control, model, and MBT1805-30 as candidates for Welch and Brown-Forsythe one-way ANOVA. Of these, variables that significantly differed among the groups (*p* < 0.05) were considered candidate biomarkers. Table 1 summarizes the 27 compounds identified by their molecular weight, formula. *p* values of Welch and Brown-Forsythe one-way analysis of variance and the *post hoc* test of Dunnett T3 are also presented in Table 1.

**TABLE 1:** Metabolites with significant differences among groups.

No	Mass	Formula	Compound	*P* ^*a*^	*P* ^*b*^	*P* ^*c*^	*P* ^*d*^	*P* ^*e*^
1	521.348141	C_26_H_52_NO_7_P	LPC (18:1/0:0)	0.015	0.003	0.292	0.044	0.010
2	408.2876	C_24_H_40_O_5_	alpha-Muricholic acid	<0.001	<0.001	0.002	0.001	0.029
3	523.363791	C_26_H_54_NO_7_P	LPC (0:0/18:0)	0.026	0.019	0.019	0.659	0.199
4	521.348141	C_26_H_52_NO_7_P	LPC (0:0/18:1)	0.019	0.009	0.119	0.016	0.631
5	161.1052	C_7_H_15_NO_3_	DL-Carnitine	0.015	0.008	0.823	0.038	0.034
6	612.152	C_20_H_23_N_6_O_12_S_2_	Glutathione oxidized	0.029	0.024	0.691	0.022	0.226
7	117.0789786	C_5_H_11_NO_2_	Betaine	0.004	0.002	0.011	0.135	0.037
8	136.0637	C_7_H_8_N_2_O	6-Methylnicotinamide	0.009	0.001	0.072	0.007	0.101
9	496.30358	C_27_H_44_O_8_	20,26-Dihydroxyecdysone	0.044	0.020	0.565	0.071	0.047
10	136.0385	C_5_H_4_N_4_O	Allopurinol	0.010	0.002	0.057	0.007	0.187
11	281.112404	C_11_H_15_N_5_O_4_	2′-O-Methyladenosine	0.03	0.035	0.096	0.081	0.990
12	499.2968	C_26_H_45_NO_6_S	Tauroursodeoxycholic acid	0.005	0.001	0.008	0.005	0.659
13	254.2	C_16_H_30_O_2_	FFA (16:1)	0.007	0.002	0.266	0.004	0.074
14	515.291674	C_26_H_45_NO_7_S	Taurohyocholic acid	0.006	0.001	0.011	0.005	0.725
15	545.348141	C_28_H_52_NO_7_P	LPC (0:0/20:3)	0.002	0.001	0.787	0.005	0.008
16	545.348141	C_28_H_52_NO_7_P	LPC (20:3/0:0)	0.002	0.001	0.787	0.005	0.008
17	125.99868	C_2_H_6_O_4_S	Isethionic acid	0.001	0.002	0.009	0.122	0.010
18	383.108	C_14_H_17_N_5_O_8_	N6-Succinyl adenosine	0.049	0.016	0.055	0.878	0.100
19	408.2876	C_24_H_40_O_5_	gamma-Muricholic acid	0.003	0.001	0.006	0.006	0.988
20	112.027	C_4_H_4_N_2_O_2_	Uracil	<0.001	<0.001	0.002	0.001	0.247
21	515.292	C_26_H_45_NO_7_S	Taurocholic acid	0.050	0.019	0.068	0.081	0.595
22	283.092	C_10_H_13_N_5_O_5_	8-Hydroxy-2-deoxyguanosine	0.021	0.007	0.246	0.021	0.187
23	283.0916685	C_10_H_13_N_5_O_5_	2-Hydroxyadenosine	0.021	0.007	0.246	0.021	0.187
24	103.026944	C_3_H_5_NO_3_	N-Formylglycine	<0.001	<0.001	0.004	0.157	<0.001
25	479.301191	C_23_H_46_NO_7_P	LPE (18:1/0:0)	0.030	0.012	0.929	0.048	0.055
26	569.348141	C_30_H_52_NO_7_P	LPC (22:5/0:0)	0.027	0.013	0.069	0.041	0.390
27	117.078979	C_5_H_11_NO_2_	N-Methyl-aminoisobutyric acid	0.026	0.009	0.039	0.819	0.052

*P* ^*a*^, *p* value of Welch test of one-way ANOVA; *P* ^*b*^, *p* value of Brown-Forsythe test of one-way ANOVA; *P* ^*c*^, *p* value of the *post hoc* test of Dunnett T3, control vs. ANIT; *P* ^*d*^, *p* value of the *post hoc* test of Dunnett T3, ANIT vs. MBT1805-30; *P* ^*e*^, *p* value of the *post hoc* test of Dunnett T3, control vs. MBT1805-30.

### Pathway Analysis

**FIGURE 4:**
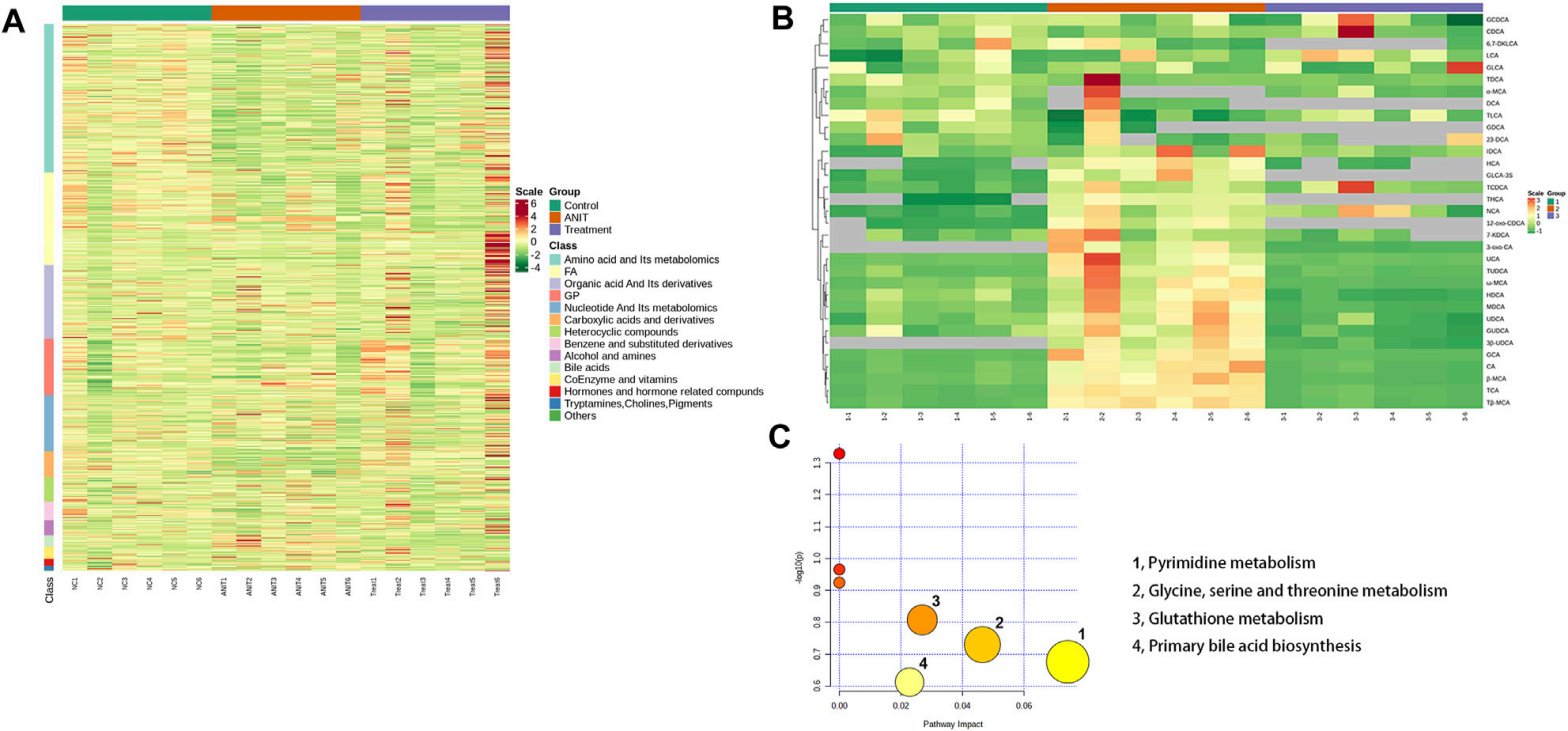
Heat map and pathways analysis among the control, ANIT, and MBT1805 treated groups. **(A)** Heat map of potential metabolites. Red in gradient presented the increases. Green in gradient presented the decreases. Samples with green were the control group (*n* = 6), samples with red were the ANIT group (*n* = 6), and samples with purple were the MBT1805-treated group. **(B)** Heat map of bile acids of bile acid-targeted metabolomic. **(C)** Bubble map of control, ANIT, and MBT1805 30 mg/kg groups in pathway analysis.

The heat map revealed changes in various types of substances among the control, model, and MBT1805-treated groups (Figure 4A). Upregulation is in red and downregulation is in green. Control, ANIT, and MBT1805-treated groups were significantly separated due to expression difference. Bile acids in the ANIT group were upregulated while reduced in the MBT1805-treated group. Figure 4B shows the heatmap of bile acids in bile acid-targeted metabolomics. MBT1805 significantly reversed ANIT-induced abnormal contents of bile acids. As shown in Figure 4C, the top four pathways were pyrimidine metabolism, glycine, serine and threonine metabolism, glutathione metabolism, and primary bile acid biosynthesis. Untargeted and bile acid-targeted metabolomics indicated that MBT1805 was effective in improving cholestasis and probably achieved by regulating bile acid metabolism.

### Quantitative Analysis of Bile Acids

**FIGURE 5:**
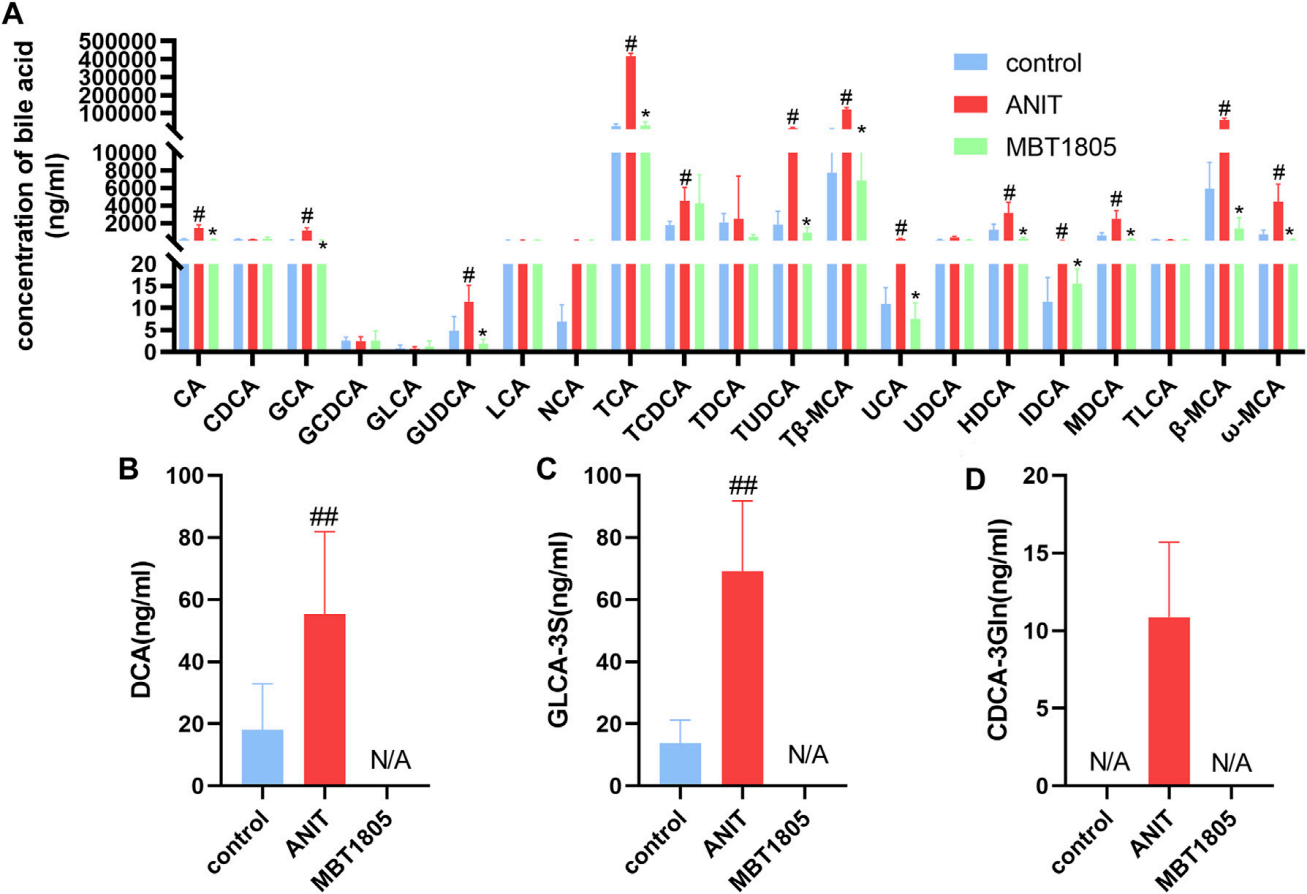
Bile acid levels of different groups. Analysis of 21 common bile acids **(A)**. Analysis of several hydrophobic bile acids, Deoxycholic acid, DCA **(B)**; Glycolithocholic acid-3-sulfate, GLCA-3S **(C)**; Chenodeoxycholic acid-3-β-D-glucuronide, CDCA-3Gln **(D)**. Control, control group; ANIT, model group; MBT1805, MBT1805 30 mg/kg treated group; #*p* < 0.05, ##*p* < 0.01, vs. control; *p < 0.05, vs. ANIT.

Metabolomic analysis revealed that the bile acid biosynthesis pathway was an essential pathway altered by MBT1805. In agreement with the results of untargeted metabolomics, the levels of conjugated bile acids, taurine-conjugated bile acids (TCA, TCDCA, TUDCA, and Tβ-MCA), and glycine-conjugated bile acids (GCA and GUDCA) were significantly elevated in the model compared with the control but significantly reduced in MBT1805-treated groups (Figure 5). In summary, bile acid metabolism was disrupted by ANIT, whereas MBT1805 treatment significantly reversed the abnormality of bile acid homeostasis.

### Expression Levels of Key Enzymes and Transporters That Regulate Bile Acid Synthesis, Detoxification, and Transport

**FIGURE 6:**
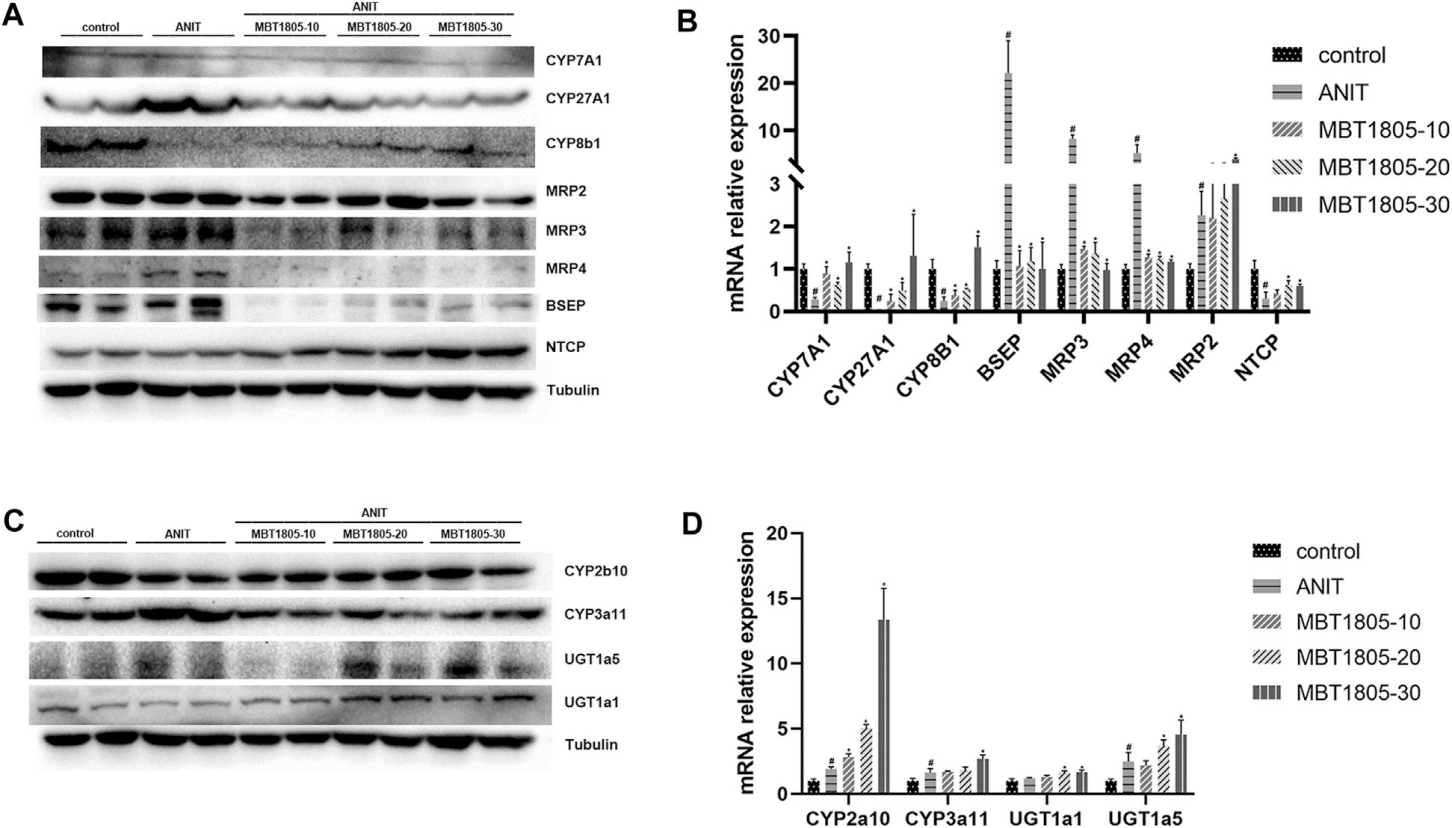
Influence of MBT1805 on the expression of bile acid synthesis enzymes and transports. Data are mean ± SD, *n* = 5. #*p* < 0.05 and ##*p* < 0.01 compared with control group; *p < 0.05 and **p < 0.01 compared with ANIT group. **(A**,**C)** Western blot; **(B**,**D)** qRT-PCR.

Primary bile acid, including CA and CDCA, is synthesis by classical and alternative pathways, which are mediated by CYP7A1 and CYP27A1, respectively (Wahlström et al., 2016). In ANIT-induced cholestasis, the synthesis of bile acid increased, especially through an alternative pathway. However, mRNA and protein expressions were inconsistent with CYP 27A1 (Figures 6A,B). We supposed that a decrease in the mRNA level of CYP27A1 was an adaptive reaction when cholestasis had formed. MBT1805 significantly reduced the expression of CYP27A1; thus, the synthesis of bile acid was interrupted. In the cholestasis model, transport of bile acid from hepatocytes to bile duct and to systemic circulation was increased, because the expressions of BSEP and multidrug resistance proteins (MRP2, MRP3, and MRP4) were evaluated. BESP and MRP2 mediate transport of bile acid from hepatocytes to the bile duct, while MRP3 and MRP4 mediate transport to the systemic circulation (Jia et al., 2018). MBT1805 decreased bile acid transport to circulation; therefore, serum indicators significantly improved. Bile acid reabsorption mediated by sodium taurocholate cotransporting polypeptide (NTCP) was inhibited in the cholestasis model and was restored in the MBT1805-treated group. Hepatic biotransformation, especially phase Ⅱ metabolic reaction, is crucial in reducing the toxicity of hydrophobic bile acid. MBT1805 could also regulate the expressions of UDP-glucuronosyltransferases, which contributed to detoxication.

## Discussion

Accumulation of toxic bile acids due to destruction or obstruction of bile ducts is the main cause of cholestasis. UDCA and OCA are currently recommended treatment options for cholestatic liver injury (Gao et al., 2020). However, up to 40% of patients with PBC have an inadequate response to UDCA treatment, whereas OCA can aggravate pruritus, which is a common symptom of cholestasis (Roberts et al., 2020; Gomez et al., 2021). PPAR agonists, used as drugs for hyperlipidemia and type2 diabetes, show some encouraging results in PBC treatment (Jones et al., 2017; Corpechot et al., 2018) and, thus, are considered potential therapeutic targets.

MBT1805 was built on resveratrol scaffolds and has a balanced PPARα/γ/δ activation effect. In this study, we aimed to assess the therapeutic effects of MBT1805 and explore the underlying mechanisms of action against ANIT-induced cholestasis. ANIT causes pathological necrosis of hepatocytes and intrahepatic bile ducts, periportal inflammation, and fibrosis in mice (Mariotti et al., 2018). We found that serum levels of biochemical indicators of liver injury (ALT and AST) significantly increased in the model compared with the control and that MBT1805 could significantly reduce the levels in a dose-dependent manner (Figures 1A,B). A similar pattern was observed for Tbil (Figure 1C). ALT, AST, and Tbil levels in the MBT1805-treated group were lower than those in the UDCA-treated group. But ALP level (Figure 1C) was lower in the UDCA-treated group. Additionally, reduced ALP was observed only in the high dose of the MBT1805-treated group. We speculated that the difference of PPARs distribution and affinity to agonist between hepatocytes and bile duct cells might provide an explanation. PPARα (Yang et al., 2019) and PPARγ (Xiang et al., 2021) have been reported to have protective effects in cholestasis by regulating bile acid metabolism in hepatocytes. In cholangiocytes, studies mainly focused on PPARγ, which played an important role in maintaining immune tolerance (Marra et al., 2005; Zhang et al., 2018). Therefore, hepatocytes and cholangiocytes could respond inconsistently to pan-PPAR agonist. Histopathological observations revealed that bile acid was highly accumulated in the gallbladder of the model, whereas the levels in the MBT1805-treated groups were similar to those in the control (Figure 2A). Besides, necrosis of the liver tissues, vacuolation, and inflammatory infiltration were observed in the model; however, no necrosis was found in the MBT1805-treated groups, whereas inflammatory infiltration was markedly reduced. Overall, the serological and histopathological results suggested that MBT1805 was an effective anticholestasis compound.

To investigate the related mechanisms of MBT1805 against cholestasis, we employed metabolomic analysis using UPLC-MS/MS, which mainly focuses on substances with molecular weights <1,000 Da. We detected 793 compounds after combining ESI^+^/ESI^−^ detection patterns and deleting any duplicates. PCA distinguished the model from the control and MBT1805-treated groups and revealed an overlap of the latter two groups. Metabolites with VIP ≥2 were selected after PLS-DA of the control, model, and MBT1805-treated groups. In total, 27 compounds were identified as significant markers (Table 1). In the model, the bile acid metabolism increased, and the phospholipid metabolism decreased. Fang et al. reported that elevated levels of lysophosphatidylcholine 18:0 activate the NF-κB/IL-6/STAT3 signaling pathway in ANIT-induced hepatotoxicity and aggravate inflammatory damage. In ANIT-induced injury, the ANIT-glutathione complex is secreted into the bile mediated by MRP2, exhausting glutathione that plays an essential role in eliminating free radicals and protecting against oxidative stress injury (Christoph G. Dietrich, 2001). We found that MBT1805 significantly reduced the levels of lysophosphatidylcholine 18:0 and oxidative glutathione and increased the level of glutathione, which could contribute to decreased hepatic inflammatory infiltration and reactive oxygen species production.

Pathway analysis identified that the top four pathways were pyrimidine metabolism, glycine, serine and threonine metabolism, glutathione metabolism, and primary bile acid biosynthesis, indicating that MBT1805 probably influences bile acid metabolism and plays an important role in cholestasis. To further explore the effects of MBT1805 on bile acid metabolism in cholestasis, we carried out bile acid target metabolomics and identified more than 20 bile acids. Cholestasis is characterized by the intrahepatic accumulation of excessive bile acids, which probably induces hepatic parenchymal cell death, bile duct proliferation, liver inflammation, and fibrosis (Cai and Boyer, 2021). Hydrophobic bile acid retention promotes oxidative stress that causes mitochondrial dysfunction, leading to liver damage (Arduini et al., 2012). In the present study, metabolomics revealed that levels of bile acids, including CA, GCA, β/ωMCA, and Tβ-MCA, were modulated by ANIT and reversed by MBT1805. In particular, some hydrophobic and toxic acid derivatives such as DCA, GLCA-3S, and CDCA-3Gln were reduced to undetectable levels in the MBT1805-treated group, as shown in Figures 5B–D. Therefore, MBT1805 effectively reduced the accumulation of toxic bile acids accumulation and their damage to hepatocytes.

Hepatic bile acid metabolism is a complex biochemical process. Hepatic cholestasis occurs when bile acid metabolism processes are abnormal, including bile acid synthesis, biotransformation, and transport. qRCR and Western blot were performed to verify the expression of key enzymes and transporters regulating bile acid synthesis, biotransformation, and transport. Herein, we found an increase of bile acid synthesis in ANIT-induced cholestasis, particularly alternative pathway of bile acid synthesis. CYP27A1 is the rate-limiting enzyme of alternative pathway (Wahlström et al., 2016), whose expression significantly increases in the model group (Figure 6A). However, results of mRNA and protein expression levels were inconsistent (Figures 6A,B), and we speculated that a decrease of mRNA expression could be an adaptive response when cholestasis had been already formed. BESP and MRP2 mediate bile acid transport from hepatocytes to bile duct, while MRP3 and MRP4 mediate bile acid transport from hepatocytes to the systemic circulation (Jia et al., 2018). In the ANIT-induced cholestasis model, transport from hepatocytes to bile duct and to systemic circulation was enhanced, and reabsorption of bile acid mediated by NTCP was inhibited, while MBT1805 significantly reversed the abnormal expression of bile acid transport. Glucuronosyltransferase enzymes play an important role in hepatic phase II metabolic reactions, which mediate the conversion of hydrophobic bile acids to hydrophilic bile acid, thus reducing bile acid toxicity (Burchell, 2003). MBT1805 could enhance bile acid biotransformation (Figures 6C,D), which contributed to relieve the toxicity of bile acid.

Hepatic steatosis accompanies cholestasis usually. We have evaluated hepatic triacylglycerol to describe hepatic cytoarchitecture. As shown in appendix (Supplementary Figure S6A), triacylglycerol level in the ANIT-treated group elevated, without significant difference. In addition, the decrease of triacylglycerol in MBT1805-treated also had no statistical difference. We speculated that ANIT-induced cholestasis was a model of acute liver injury. Steatosis could be present if damage persisted. In addition, mRNA expressions of PPARα, PPARβ/δ, and PPARγ were detected. As we see in Supplementary Figure S6B, mRNA expressions of PPARα, PPARβ/δ, and PPARγ were significantly elevated.

In summary, MBT1805 significantly improved cholestasis induced by ANIT. Hepatic nontargeted and bile acid-targeted metabolomic analysis suggested that MBT1805 could alleviate cholestasis through regulating bile acid metabolic pathway. Results of qRT-PCR and Western blot indicated that MBT1805 significantly inhibited synthesis of bile acid and enhanced phase II metabolic reactions.

## Conclusion

Our study focused on the anticholestatic effects of the novel PPARα/γ/δ agonist MBT1805. Serum biochemistry and liver histopathology indicated that MBT1805 effectively protected against cholestasis. Untargeted metabolomics combined with bile acid-targeted metabolomics revealed that the abnormal primary bile acid biosynthesis caused by ANIT was recovered by MBT1805. We also verified the key enzymes and transporters involved in the bile acid metabolism pathway, showing that MBT1805 significantly reversed the abnormal synthesis, metabolism, and transport of bile acids. In summary, MBT1805 is a potential candidate drug for cholestasis.

## Data Availability

The original contributions presented in the study are included in the article/Supplementary Material, further inquiries can be directed to the corresponding authors.
