# Supplementary material for: Metabolomic Analysis Reveals the Therapeutic Effects of MBT1805, a Novel Pan-Peroxisome Proliferator-Activated Receptor Agonist, on α-Naphthylisothiocyanate-Induced Cholestasis in Mice
Source: Front Pharmacol. 2021 Oct 29;12:732478. doi: 10.3389/fphar.2021.732478 (PMC8585842; doi:10.3389/fphar.2021.732478)
Supplement: Supplementary file 1 [file DataSheet1.docx]

Supplementary Material

**S1**

**HPLC conditions for untargeted metabolomic**

The sample extracts were analyzed using an LC-ESI-MS/MS system (UPLC, ExionLC AD, https://sciex.com.cn/; MS, QTRAP® System, https://sciex.com/). The analytical conditions were as follows, UPLC: column, Waters ACQUITY UPLC HSS T3 C18 (1.8 µm, 2.1 mm*100 mm); column temperature, 40 °C; flow rate, 0.4 mL/min; injection volume, 2μL; solvent system, water (0.1% formic acid): acetonitrile (0.1% formic acid); gradient program, 95:5 V/V at 0 min, 10:90 V/V at 10.0 min, 10:90 V/V at 11.0 min, 95:5 V/V at 11.1 min, 95:5 V/V at 14.0 min.

**ESI-QTRAP-MS/MS**

LIT and triple quadrupole (QQQ) scans were acquired on a triple quadrupole-linear ion trap mass spectrometer (QTRAP), QTRAP® LC-MS/MS System, equipped with an ESI Turbo Ion-Spray interface, operating in positive and negative ion mode and controlled by Analyst 1.6.3 software (Sciex). The ESI source operation parameters were as follows: source temperature 500 °C; ion spray voltage (IS) 5500 V (positive), -4500 V (negative); ion source gas I (GSI), gas II (GSII), curtain gas (CUR) was set at 55, 60, and 25.0 psi, respectively; the collision gas (CAD) was high. Instrument tuning and mass calibration were performed with 10 and 100 μmol/L polypropylene glycol solutions in QQQ and LIT modes, respectively. A specific set of MRM transitions were monitored for each period according to the metabolites eluted within this period.

**S2**

**HPLC conditions for bile acid-targeted metabolomic**

The sample extracts were analyzed using an LC-ESI-MS/MS system (UHPLC， ExionLC™ AD， https://sciex.com.cn/; MS, Applied Biosystems 6500 Triple Quadrupole, https://sciex.com.cn/). The analytical conditions were as follows, HPLC: column, Waters ACQUITY UPLC HSS T3 C18 (100 mm×2.1 mm i.d.，1.8 µm); solvent system, water with 0.01% acetic acid and 5mmol/L ammonium acetate (A), acetonitrile with 0.01% acetic acid (B); The gradient was optimized at 5% to 40%B in 0.5 min, then increased to 50% B in 4 min, then increased to 75% B in 3 min, and then 75% to 95% in 2.5min, washed with 95%B for 2min ,finally ramped back to 5% B (12-14 min); flow rate, 0.35 mL/min; temperature, 40°C; injection volume: 3 μL. The effluent was alternatively connected to an ESI-triple quadrupole-linear ion trap (QTRAP)-MS.

**ESI-MS/MS**

The sample extracts were analyzed using an LC-ESI-MS/MS system (UHPLC, ExionLC™ AD， https://sciex.com.cn/; MS, Applied Biosystems 6500 Triple Quadrupole, https://sciex.com.cn/). The analytical conditions were as follows, HPLC: column, Waters ACQUITY UPLC HSS T3 C18 (100 mm×2.1 mm i.d.，1.8 µm); solvent system, water with 0.01% acetic acid and 5mmol/L ammonium acetate (A), acetonitrile with 0.01% acetic acid (B); The gradient was optimized at 5% to 40%B in 0.5 min, then increased to 50% B in 4 min, then increased to 75% B in 3 min, and then 75% to 95% in 2.5min, washed with 95%B for 2min ,finally ramped back to 5% B (12-14 min); flow rate, 0.35 mL/min; temperature, 40°C; injection volume: 3 μL. The effluent was alternatively connected to an ESI-triple quadrupole-linear ion trap (QTRAP)-MS.

**S3. Primers sets for quantitative RT-PCR analysis.**

|  | species | Former sequence | Reverse sequence |
| --- | --- | --- | --- |
| β-actin | Mouse | CATTGCTGACAGGATGCAGAAGG | TGCTGGAAGGTGGACAGTGAGG |
| CYP27A1 | Mouse | TCAGGAGACCATCGGCACCTTT | CCAGTCACTTCCTTGTGCAAGG |
| CYP7A1 | Mouse | CACCATTCCTGCAACCTTCTGG | ATGGCATTCCCTCCAGAGCTGA |
| CYP8B1 | Mouse | CATGAAGGCTGTGCGTGAGGAA | CATCACGCTGTCCAACACTGGA |
| BSEP | Mouse | CCTTGGTAGAGAAGAGGCGACA | ATGGCTACCCTTTGCTTCTGCC |
| MRP3 | Mouse | ACTTCCTCCGAAACTACGCACC | GCTGGCTCATTGTCTGTCAGGT |
| MRP4 | Mouse | CACTCAGGAAACGAACCTTCTCC | TTGCACTGCCTGCGTGTTCTCT |
| MRP2 | Mouse | TACCAGCGAGTTATCGAAGCGTG | TGCTTCTGACCGCCACTGAGAT |
| NTCP | Mouse | CCTGATGCCTTTCACTGGCTTC | GGATGGTAGAACAGAGTTGGACG |
| UGT1a1 | Mouse | GCTTCTTCCGTACCTTCTGTTG | GCTGCTGAATAACTCCAAGCAT |
| UGT1a5 | Mouse | TTGGGGGCATAAACTGTGTT | GACCATGGATCCCAAAGAGA |
| CYP3a11 | Mouse | ACAGCACTGGTCAGAGCCTGAA | GAGAGCAAACCTCATGCCAAGG |
| CYP2b10 | Mouse | TGCTGTCGTTGAGCCAACC | CCACTAAACATTGGGCTTCCT |
| PPARα | Mouse | ACCACTACGGAGTTCACGCATG | GAATCTTGCAGCTCCGATCACAC |
| PPARβ/δ | Mouse | GGACCAGAACACACGCTTCCTT | CCGACATTCCATGTTGAGGCTG |
| PPARγ | Mouse | GTACTGTCGGTTTCAGAAGTGCC | ATCTCCGCCAACAGCTTCTCCT |

**S4. Antibodies used in western blot.**

| **Antibody** | **Brand and CAT. No** | **Host** | **Dilution** |
| --- | --- | --- | --- |
| CYP27A1 | Abcam, ab126785 | Rabbit | 1:1000 |
| CYP7A1 | Bioss, bs-21430R | Rabbit | 1:500 |
| CYP8B1 | Absin, abs135064 | Rabbit | 1:1000 |
| MRP4 | Absin, abs136441 | Rabbit | 1:1000 |
| MRP2 | Absin, abs145816 | Rabbit | 1:1000 |
| MRP3 | Cell signaling Technology, 39909S | Rabbit | 1:1000 |
| BSEP | Absin, abs138798 | Rabbit | 1:1000 |
| NTCP | Santa, sc-518115 | Mouse | 1:1000 |
| CYP2b10 | Santa, sc-73546 | Mouse | 1:1000 |
| CYP3a11 | Santa, sc-70903 | Mouse | 1:1000 |
| UGT1a1 | Absin, abs134323 | Rabbit | 1:1000 |
| UGT1a5 | Novus, NBP1-91336 | Rabbit | 1:500 |
| Tubulin | Abmart, M30109S | Mouse | 1:1000 |
| Anit-rabbit IgG, HRP-linked antibody | Cell signaling technology, 7074P2 | Goat | 1:2000 |
| Anti-mouse IgG,HRP-linked antibody | Cell signaling, technology, 7076P2 | Goat | 1:2000 |

**
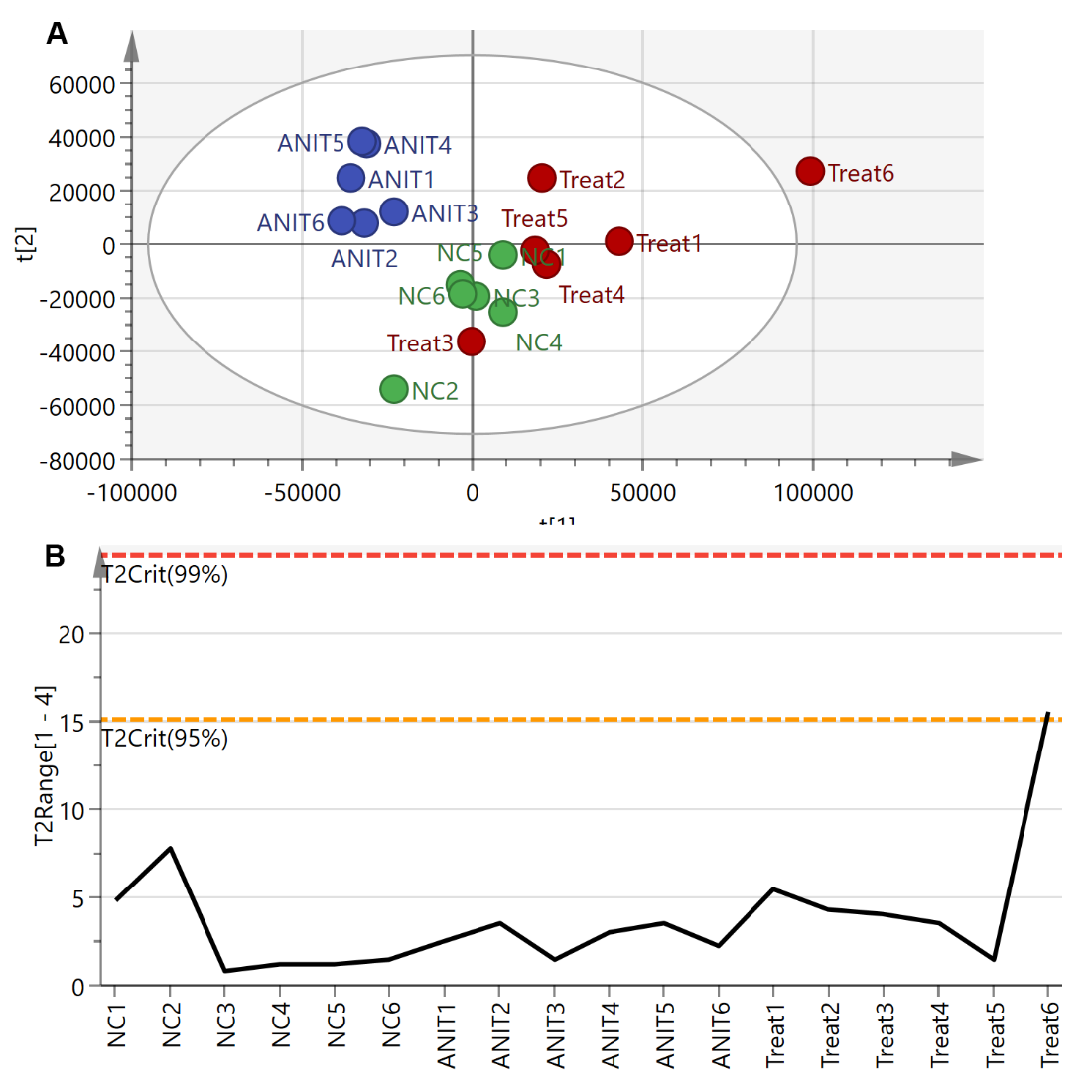
S5.** Principal component analysis of 18 samples. (A) Score plots; (B) Hotelling’s T2 Range.

**S6.** Hepatic triacylglycerol (TG) detection (A) and mRNA expression of PPAR-alpha, beta, and gamma (B). #, p<0.05, vs. control; *, p<0.05, vs ANIT. (n=5 for each group) .

**S6A**


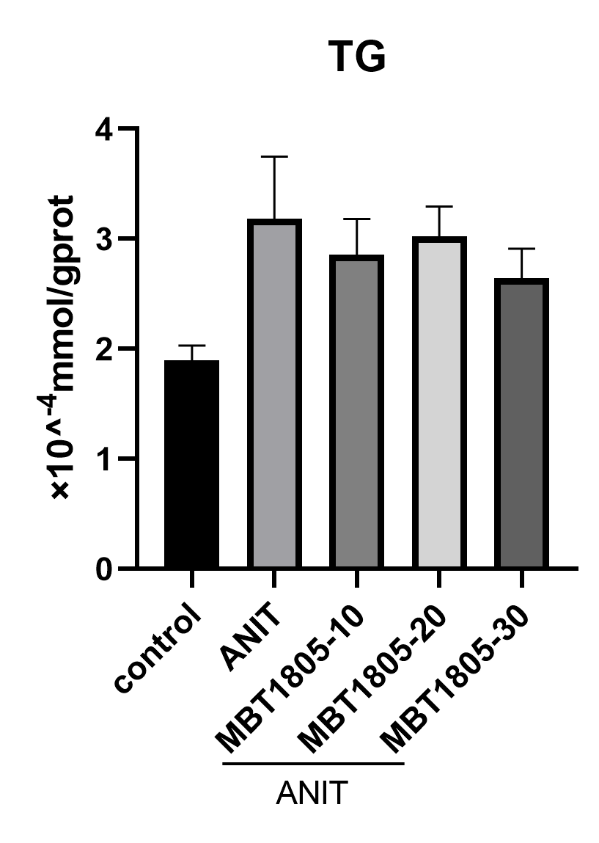


**S6B**

**
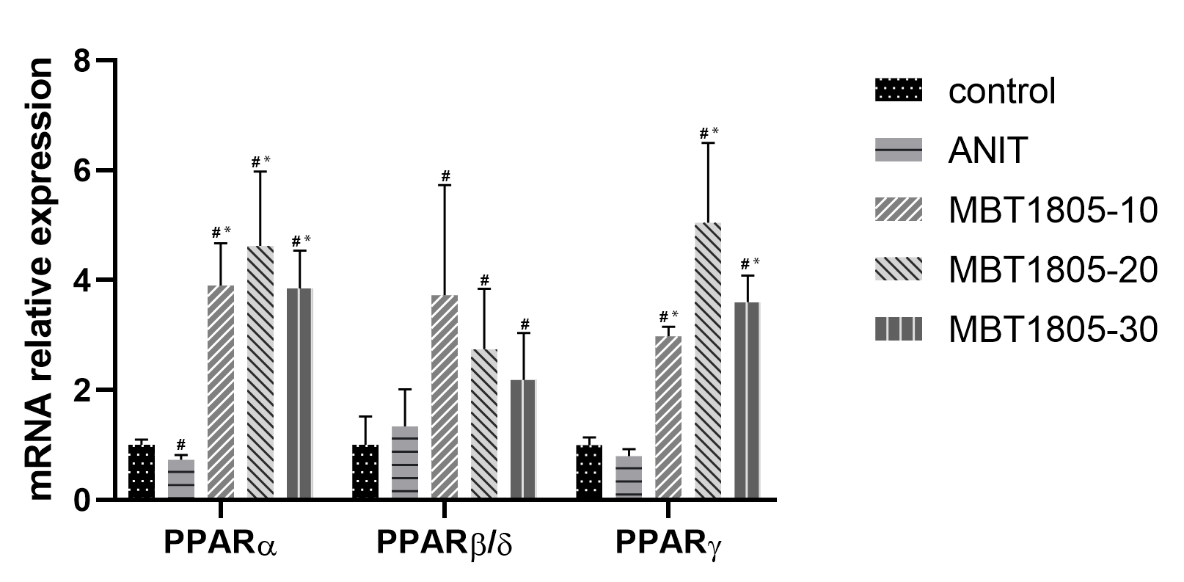
**
